# Colonization with ubiquitous protist *Blastocystis* ST1 ameliorates DSS-induced colitis and promotes beneficial microbiota and immune outcomes

**DOI:** 10.1038/s41522-023-00389-1

**Published:** 2023-04-25

**Authors:** Lei Deng, Lukasz Wojciech, Chin Wen Png, Yan Qin Dorinda Kioh, Geok Choo Ng, Eric Chun Yong Chan, Yongliang Zhang, Nicholas R. J. Gascoigne, Kevin Shyong Wei Tan

**Affiliations:** 1 Laboratory of Molecular and Cellular Parasitology, Healthy Longevity Translational Research Programme and Department of Microbiology and Immunology, Yong Loo Lin School of Medicine, National University of Singapore, 5 Science Drive 2, Singapore, 117545 Singapore; 2 Immunology Translational Research Programme and Department of Microbiology and Immunology, Yong Loo Lin School of Medicine, National University of Singapore, 5 Science Drive 2, Singapore, 117545 Singapore; 3 Department of Pharmacy, Faculty of Science, National University of Singapore, 18 Science Drive 4, Singapore, 117559 Singapore

**Keywords:** Microbiome, Cellular microbiology, Microbial ecology

## Abstract

*Blastocystis* is a species complex that exhibits extensive genetic diversity, evidenced by its classification into several genetically distinct subtypes (ST). Although several studies have shown the relationships between a specific subtype and gut microbiota, there is no study to show the effect of the ubiquitous *Blastocystis* ST1 on the gut microbiota and host health. Here, we show that *Blastocystis* ST1 colonization increased the proportion of beneficial bacteria *Alloprevotella* and *Akkermansia*, and induced Th2 and Treg cell responses in normal healthy mice. ST1-colonized mice showed decreases in the severity of DSS-induced colitis when compared to non-colonized mice. Furthermore, mice transplanted with ST1-altered gut microbiota were refractory to dextran sulfate sodium (DSS)-induced colitis via induction of Treg cells and elevated short-chain fat acid (SCFA) production. Our results suggest that colonization with *Blastocystis* ST1, one of the most common subtypes in humans, exerts beneficial effects on host health through modulating the gut microbiota and adaptive immune responses.

## Introduction

The human gut comprises thousands of diverse microbes including bacteria, fungi, archaea, viruses, and single cell eukaryotes (SCEs)^1^. Although numerous studies have focused on the effect of gut bacteria on host health and diseases over the past decades, the role of SCEs in the gut ecosystem has only recently begun to be recognized^2^. *Blastocystis* is a common gut SCE found in humans and a wide range of animals with an estimated more than 1 billion people colonized worldwide^3^. It mainly colonizes the host’s large intestine, where it has a close relationship with gut microbiota^4^. The relationship between *Blastocystis* and gut microbiota is an emerging area of research interest, and multiple studies have shown that *Blastocystis* colonization is asymptomatic and often associated with healthy gut microbiota^5–7^. However, other studies showed *Blastocystis* was related to decreased cognitive function in humans and colonic hypersensitivity in animal models^8,9^. The differences observed in previous studies could be attributed to the distinct characteristics of the various *Blastocystis* subtypes, resulting in differential effects on host health^10^.

To date, at least 30 subtypes have been identified across humans and animals based on the variations within sequences of the small subunit (SSU) rRNA gene, and more subtypes are emerging^11^. *Blastocystis* subtypes were also considered separate species that exhibited distinct biological features^12^. For example, considerable differences in the number of genomic features like DNA base composition, genome size, and the number of genes and introns were observed between ST1, ST4, and ST7^13^. *Blastocystis* ST7 can elevate the expression of pro-inflammatory cytokines interleukin (IL)-6, IL-1β, and tumor necrosis factor-alpha (TNFα) in murine macrophages, while *Blastocystis* ST4 did not show such an effect^14^. Besides, *Blastocystis* ST3 and ST4 colonization showed inverse correlations to the proportion of beneficial bacteria *Akkermansia* in a large cohort in Europe^7^. *Akkermansia* may play crucial roles in the prevention of several metabolic diseases, such as obesity, type 2 diabetes or hypertension^15^.

It has been reported that *Blastocystis* ST4 colonization triggers potent Th2 and Treg immune responses, which help experimentally colonized mice recover faster from experimentally-induced colitis^16^. Similarly, long-term colonization with *Blastocystis* ST3 could promote faster recovery from colitis and decrease the expression of TNFα and IL-1β in dinitrobenzene sulfonic acid (DNBS)-induced colitis mice^17^. In contrast, *Blastocystis* ST7 infection worsens the severity of colitis induced by dextran sulfate sodium (DSS) in a mouse model^18^. These data suggest that it is necessary to investigate the effects of *Blastocystis* on host health at the subtype level. In this study, we investigated a specific subtype, ST1, one of the most common subtypes in humans worldwide^19^. We firstly investigated the effect of *Blastocystis* ST1 colonization on gut microbiota and adaptive immune responses, and then further explored the role of ST1-altered gut microbiota in the development of experimentally-induced colitis. Our data provide valuable insights into host-parasite interactions.

## Results

### Effects of *Blastocystis* ST1 colonization on normal healthy mice

**Fig. 1:**
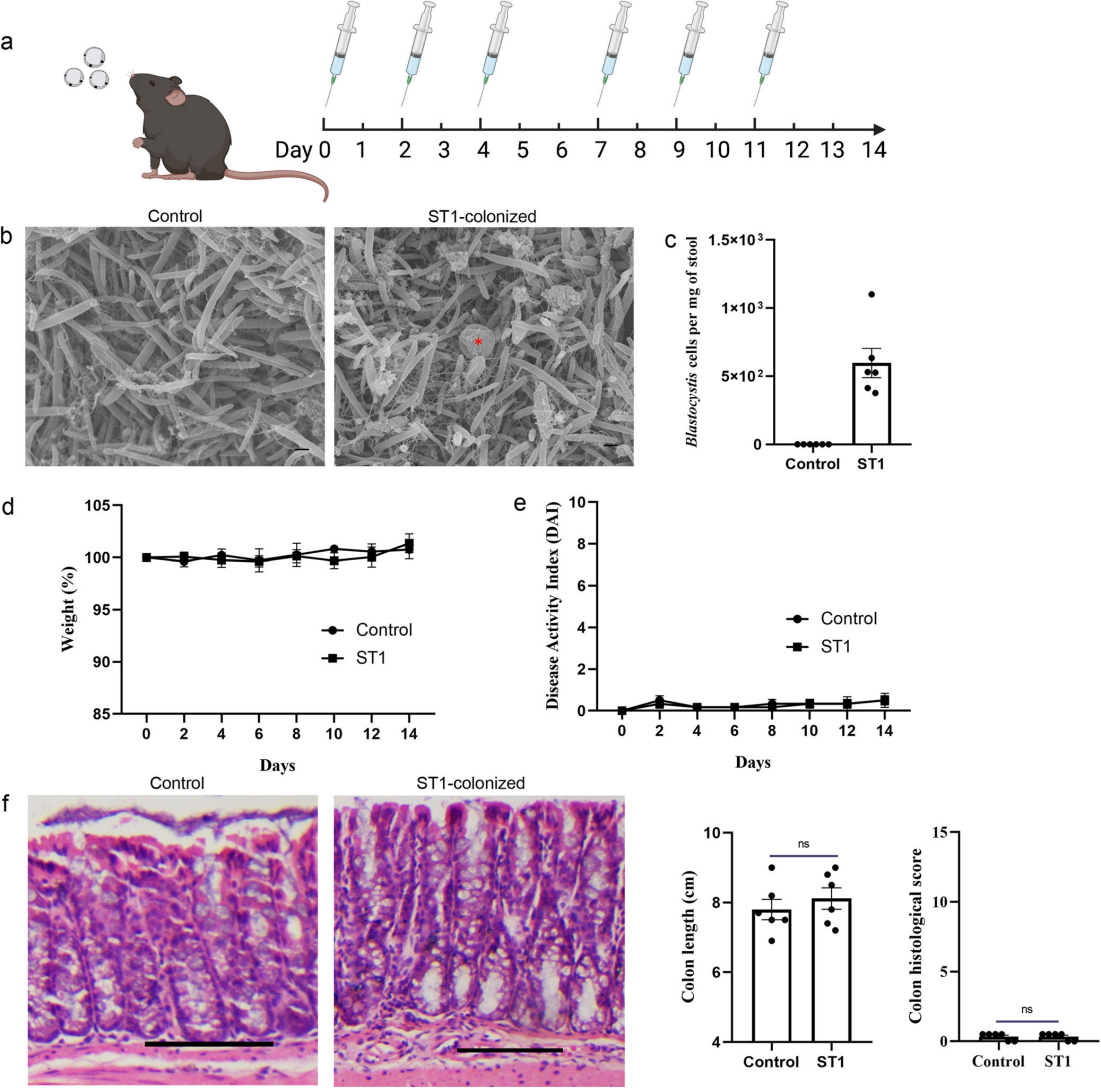
*Blastocystis* ST1 colonization does not cause any harmful effects on normal healthy mice. **a** Experimental design. **b** Scanning electron microscopy of colon and cecum tissues from ST1-colonized and un-colonized mice, ST1 are indicated with a red asterisk (*). Scale bar = 1 μm. **c** *Blastocystis* ST1 cells per milligram of stool in ST1-colonized mice. Weight changes (**d**) and DAI (**e**) between control and ST1-colonized mice. **f** Representative micrographs of H&E-stained colon sections, colon length, and colonic histological scores from un-colonized and ST1-colonized mice. Scale bar = 100 μm. Data are shown as the mean ± SEM. Significance was determined by two-sided unpaired Student’s *t* test (ns non-significant).

Multiple epidemiologic studies have shown that *Blastocystis* ST1 is one of the most common subtypes in humans^19^, while the effect of ST1 colonization on host health remains largely unknown. The mice were orally gavaged with *Blastocystis* ST1 for two weeks (Fig. 1a). *Blastocystis* colonization was confirmed by SEM and qPCR (Fig. 1b, c). Clinical manifestations were assessed by evaluating weight changes and DAI. There were no significant changes in the weight and DAI of mice colonized with ST1 compared to non-colonized mice (Fig. 1d, e). Similarly, ST1-colonized mice did not show any colonic histological damage and the colon lengths were comparable to control mice (Fig. 1f). These data suggest that ST1 colonizes asymptomatically in normal healthy mice, which is similar to the effects of *Blastocystis* ST4^16^.

### Effect of *Blastocystis* ST1 colonization on gut bacterial composition

**Fig. 2:**
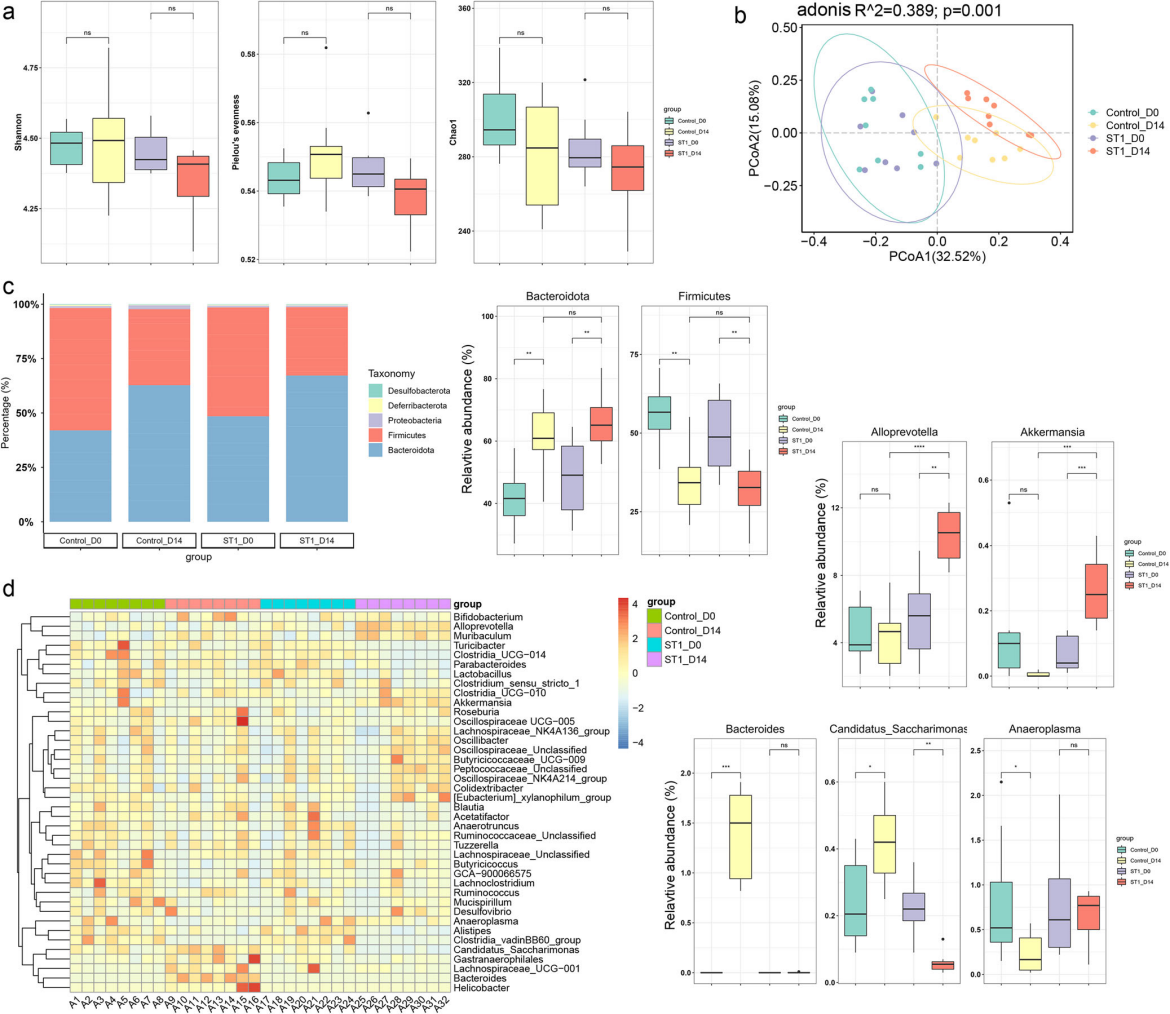
*Blastocystis* ST1 colonization alters gut microbiota compositions. **a** Alpha diversity was measured by Shannon, Pielou’s evenness, and Chao1 indices (*n* = 8 for each group). **b** PCoA of fecal gut microbiota in ST1-colonized and un-colonized mice. **c** Relative abundance of the top 5 phyla. **d** Heatmap of the 40 most abundant genera and those genera showing significant differences in *Blastocystis*-free and ST1-colonized mice. Data are presented as boxplots, with the center line representing the median, the boundary of the whiskers representing the minimum and maximum values of the dataset, and the boundary of the box representing the 25th and 75th percentile of the dataset (**a**, **c**, **d**). Significance was determined using the Wilcoxon rank-sum test. (ns non-significant; *P < 0.05; **P < 0.01; ***P < 0.001; and ****P < 0.0001).

Next, we performed 16 S rRNA gene sequencing on fecal samples from ST1-colonized and control mice to determine the effect of *Blastocystis* ST1 colonization on the gut bacterial community. The rarefaction curves showed sufficient sequencing depth was obtained for further diversity analysis (Supplementary Fig. 1a). There were no significant changes in the alpha diversity based on Shannon, Pielou’s evenness, and Chao1 indices measurements, when compared to the baseline in both ST1-colonized and control mice (Fig. 2a). At the start of the experiment (day 0), PCoA plots showed ST1-colonized and control mice clustered together, suggesting those mice showed similar microbiota composition before *Blastocystis* ST1 colonization (*P* > 0.05, Fig. 2b and Supplementary Table 1). Notably, we observed significant changes in the gut microbial compositions in both ST1-colonized and control groups by PERMANOVA analysis (*P* < 0.05, Fig. 2b and Supplementary Table 1). The fluctuations observed in the control group could be attributed to the complex and dynamic nature of the gut microbiota, which is known to change over time^20^. At the phylum level, the abundance of *Bacteroidota* was elevated in both ST1-colonized and non-colonized groups, while the proportion of Firmicutes was found to be decreased in these groups (Fig. 2c). At the genus level, *Alloprevotella* and *Akkermansia* were significantly enriched in ST1-colonized mice (Fig. 2d). *Alloprevotella* are SCFA-producing bacteria and are positively correlated with the anti-inflammatory cytokine IL-10^21,22^. It has also been determined that the administration of *Akkermansia muciniphila* can ameliorate DSS-induced colitis in mice^23^, and increase mucus production to prevent intestinal inflammation^24^. In contrast, the level of *Candidatus Saccharimonas* and *Bacteroides* was significantly higher in control mice when compared to the baseline, and the commensal bacteria *Anaeroplasma* declined in un-colonized mice (Fig. 2d). *Muribaculum*, *Alistipes*, and *Clostridia vadinBB60* group showed similar changes in both ST1-colonized and non-colonized mice (Supplementary Fig. 1b). Similarly, LefSe analysis also showed that the *Bacteroides* and *Alloprevotella* were enriched in non-colonized and ST1-colonized mice respectively (Supplementary Fig. 1c). Altogether, our data showed *Blastocystis* ST1 colonization could alter the gut microbiota composition and was positively associated with the beneficial bacteria *Alloprevotella* and *Akkermansia*.

### Effect of *Blastocystis* ST1 colonization on colonic T helper cells

**Fig. 3:**
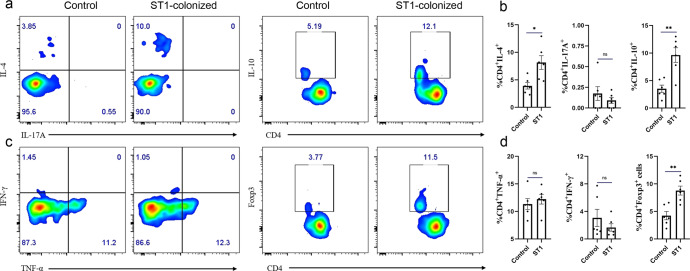
*Blastocystis* ST1 colonization induces accumulation of Th2 and Treg cells in the colonic lamina propria. **a** Colored contour plots show staining for IL-4, IL-10, and IL-17A within CD4^+^ cells. **b** Bar charts show the percentage of IL-4, IL-10, and IL-17A expressing CD4^+^ T cells. **c** Colored contour plots show staining for IFN-γ, TNF-α, and Foxp3 within CD4^+^ cells. **d** Bar charts show the percentage of IFN-γ, TNF-α, and Foxp3-expressing CD4^+^ T cells. Data are shown as the mean ± SEM. Significance was determined by two-sided unpaired Student’s *t* test (ns non-significant; *P < 0.05; **P < 0.01).

Host adaptive immune responses play a crucial role in maintaining intestinal homeostasis, and inappropriate T-cell responses against the microbiota are implicated in the pathogenesis of inflammatory bowel disease (IBD)^25^. We next investigated whether ST1 could also alter the functions of colonic T helper cells during colonization. Interestingly, the expansion of IL-4-producing CD4^+^ Th2 cells and IL-10-producing CD4^+^ Treg cells was observed in ST1-colonized mice (Fig. 3a, b). Forkhead box P3 (Foxp3) is a crucial regulator of the development and function of Treg cells^26^. We also observed an elevated proportion of Foxp3-expressing CD4^+^ T cells in the ST1-colonized mice (Fig. 3c, d). The accumulation of Th2 and Treg cells is commonly found in extracellular parasite infection, which plays a significant role in immunity, tissue repair, and tolerance of the parasite at tissue sites^27^. In contrast, the percentage of IFNγ-, and TNF-α-producing CD4^+^ Th1 cells and IL-17A-producing CD4^+^ Th17 cells did not expand in both ST1-colonized and control mice (Fig. 3a–d). These results were consistent with our previous report that *Blastocystis* ST4 colonization promotes Th2 and Treg cell differentiation to maintain immune homeostasis in normal healthy mice^16^.

### Effect of *Blastocystis* ST1 colonization on the severity of DSS-induced colitis

**Fig. 4:**
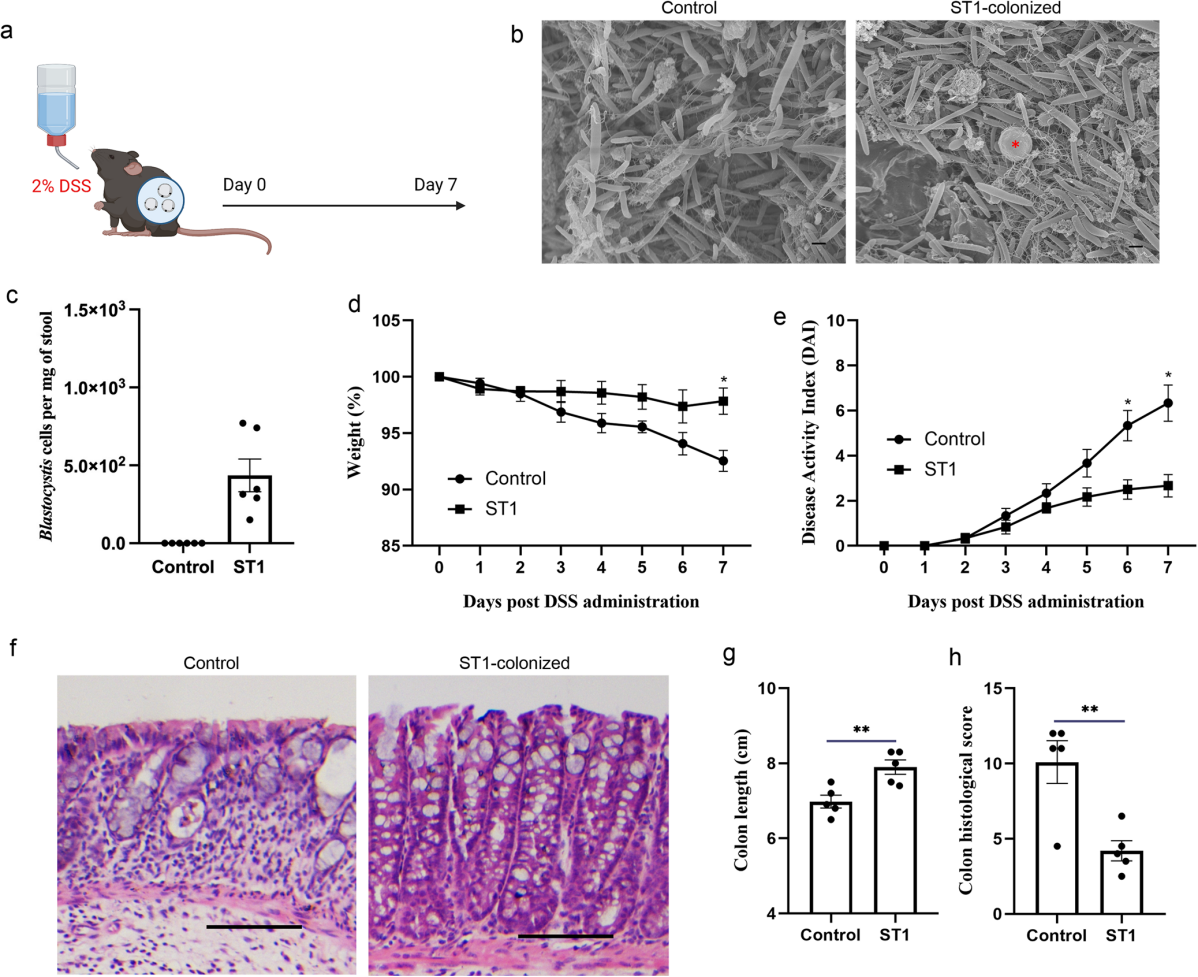
*Blastocystis* ST1 colonization exerts a protective effect on DSS-induced colitis. **a** Experimental design. **b** Scanning electron microscopy of colon and cecum tissues from ST1-colonized and un-colonized mice, ST1 are indicated with a red asterisk (*). Scale bar = 1 μm. **c** *Blastocystis* ST1 cells per milligram of stool in ST1-colonized mice. Weight changes (**d**) and DAI (**e**) between ST1-colonized and un-colonized mice during DSS treated for 7 days. **f** Representative micrographs of H&E-stained colon sections from un-colonized and ST1-colonized mice. Colon length (**g**) and colonic histological (**h**) in un-colonized and ST1-colonized group. Scale bar = 100 μm. Data are shown as the mean ± SEM. Significance was determined by two-sided unpaired Student’s *t* test (*P < 0.05; **P < 0.01).

The gut microbiota is believed to contribute to the pathogenesis of several diseases, such as IBD^28,29^. We then sought to determine whether changes in gut microbiota induced by *Blastocystis* ST1 were correlated with susceptibility to experimentally-induced colitis. ST1-colonized mice were treated with 2% DSS for one week to develop colitis (Fig. 4a). *Blastocystis* ST1 colonization status was assessed using SEM and qPCR, which indicated the presence and the number of *Blastocystis* ST1 cells in the mouse intestine respectively (Fig. 4b, c). Notably, ST1-colonized mice showed lower severity of colitis with less weight loss and lower DAI than control mice (Fig. 4d, e). The results of the colon histological examinations indicated that un-colonized mice experienced significant crypt disruption and infiltration of inflammatory cells (Fig. 4f). Similarly, ST1-colonized mice exhibited significantly longer colon length and lower colonic histological damage scores than un-colonized mice (Fig. 4g, h). These results indicated that ST1 colonization exerts a protective effect on DSS-induced colitis.

### The protective effect against DSS-induced colitis associated with Th2 and Treg immune responses

**Fig. 5:**
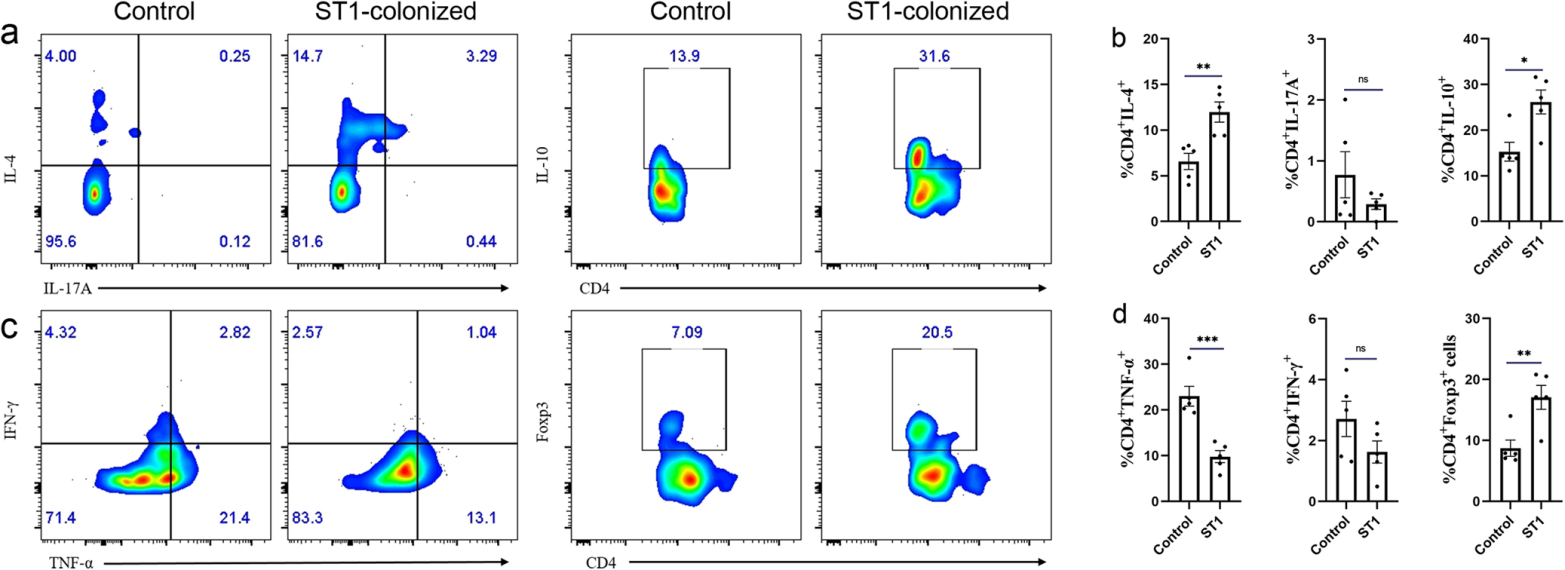
*Blastocystis* ST1 prevents experimental colitis associated with the accumulation of the Th2 and Treg cells. **a** Colored contour plots show staining for IL-4, IL-10, and IL-17A within CD4^+^ cells. **b** Bar charts show the percentage of IL-4, IL-10, and IL-17A expressing CD4^+^ T cells. **c** Colored contour plots show staining for IFN-γ, TNF-α, and Foxp3 within CD4^+^ cells. **d** Bar charts show the percentage of IFN-γ, TNF-α, and Foxp3-expressing CD4^+^ T cells. Data are shown as the mean ± SEM. Significance was determined by two-sided unpaired Student’s *t* test (ns non-significant; *P < 0.05; **P < 0.01; ***P < 0.001).

Several studies have shown that infection with helminth prevents experimental colitis by inducing the accumulation of Th2 and Treg cells^30,31^. We next investigated whether the Th2 and Treg cells are involved in the protective effects of DSS-induced colitis. Colonization with *Blastocystis* ST1 increases the production of IL-4, IL-10, and the proportion of Foxp3^+^CD4^+^ T cells (Fig. 5a, b). In contrast, the control mice showed a substantial increase in the percentage of pro-inflammatory cytokine TNF-α-producing T cells (Fig. 5c, d). IL17-A-producing CD4^+^ T cells in the colonic mucosa were comparable between ST1-colonized and control mice (Fig. 5a, b). These data suggest that colonization with *Blastocystis* ST1 prevents experimental colitis associated with the accumulation of the Th2 and Treg cells.

### Effect of *Blastocystis* ST1-altered gut microbiota on the severity of DSS-induced colitis

**Fig. 6:**
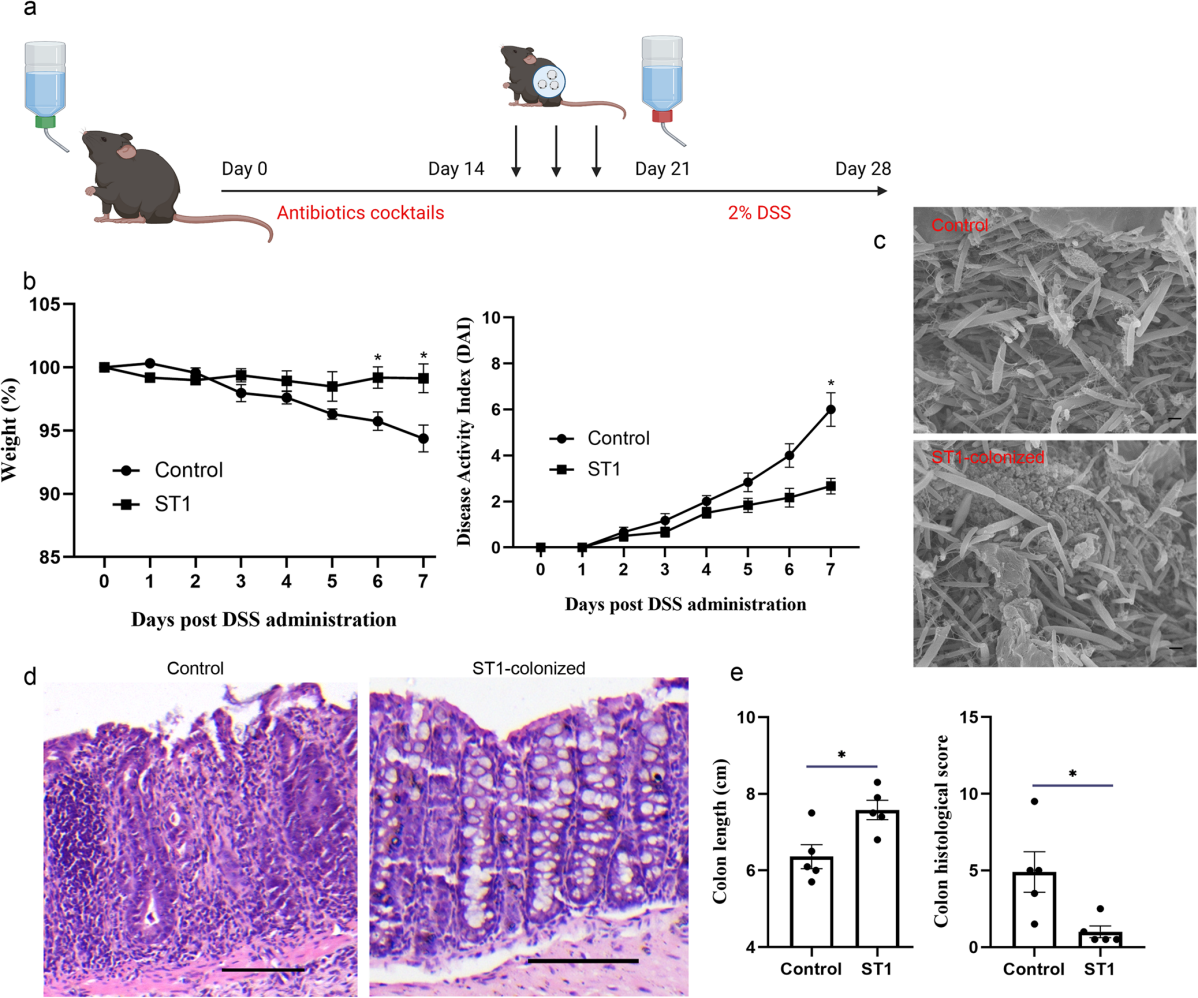
*Blastocystis* ST1-altered gut microbiota exerts a protective effect on DSS-induced colitis. **a** Experimental design. **b** Weight changes and DAI between mice with ST1-altered gut microbiota and control donor during DSS treated for 7 days. **c** SEM showed no *Blastocystis* cells in both mice with ST1-altered gut microbiota and control donor. Scale bar = 1 μm. **d** Representative micrographs of H&E-stained colon sections from mice with control donor and ST-1 altered gut microbiota. **e** Colon length and colon histology in mice with control donor and ST-1 altered gut microbiota. Scale bar = 100 μm. Data are shown as the mean ± SEM. Significance was determined by two-sided unpaired Student’s *t* test (*P < 0.05).

To determine whether *Blastocystis* ST1-altered gut microbiota can influence the susceptibility of DSS-induced colitis, the mice were treated with a cocktail of the antibiotic for two weeks and then were orally gavaged with *Blastocystis*-altered gut microbiota (Fig. 6a). The fecal samples used for FMT were taken from both ST1-colonized and control mice (Fig. 1a). To disrupt the live *Blastocystis* ST1 cells, the samples were subjected to a freeze-thaw process (Supplementary Fig. 2). Mice that received fecal transplants from the control donor treated with DSS showed progressive weight loss and higher DAI compared to ST1-altered gut microbiota recipient mice (Fig. 6b). SEM and feces culturing did not reveal *Blastocystis* cells in the caecum or colon, suggesting the protective effects are associated with ST1-altered gut microbiota (Fig. 6c). Examination of colon tissue from DSS treated recipient mice found that control mice exhibited a higher degree of inflammation and tissue damage than the ST1-altered gut microbiota recipient mice treated with DSS (Fig. 6d). A shorter colon length was observed in control mice when compared to ST1-altered gut microbiota recipient mice (Fig. 6e). These results suggest that transplantation of *Blastocystis*-altered gut microbiota could exert the similar protective effects on the severity of colitis.

### Effect of *Blastocystis* ST1-altered gut microbiota on T helper cells and SCFA production

**Fig. 7:**
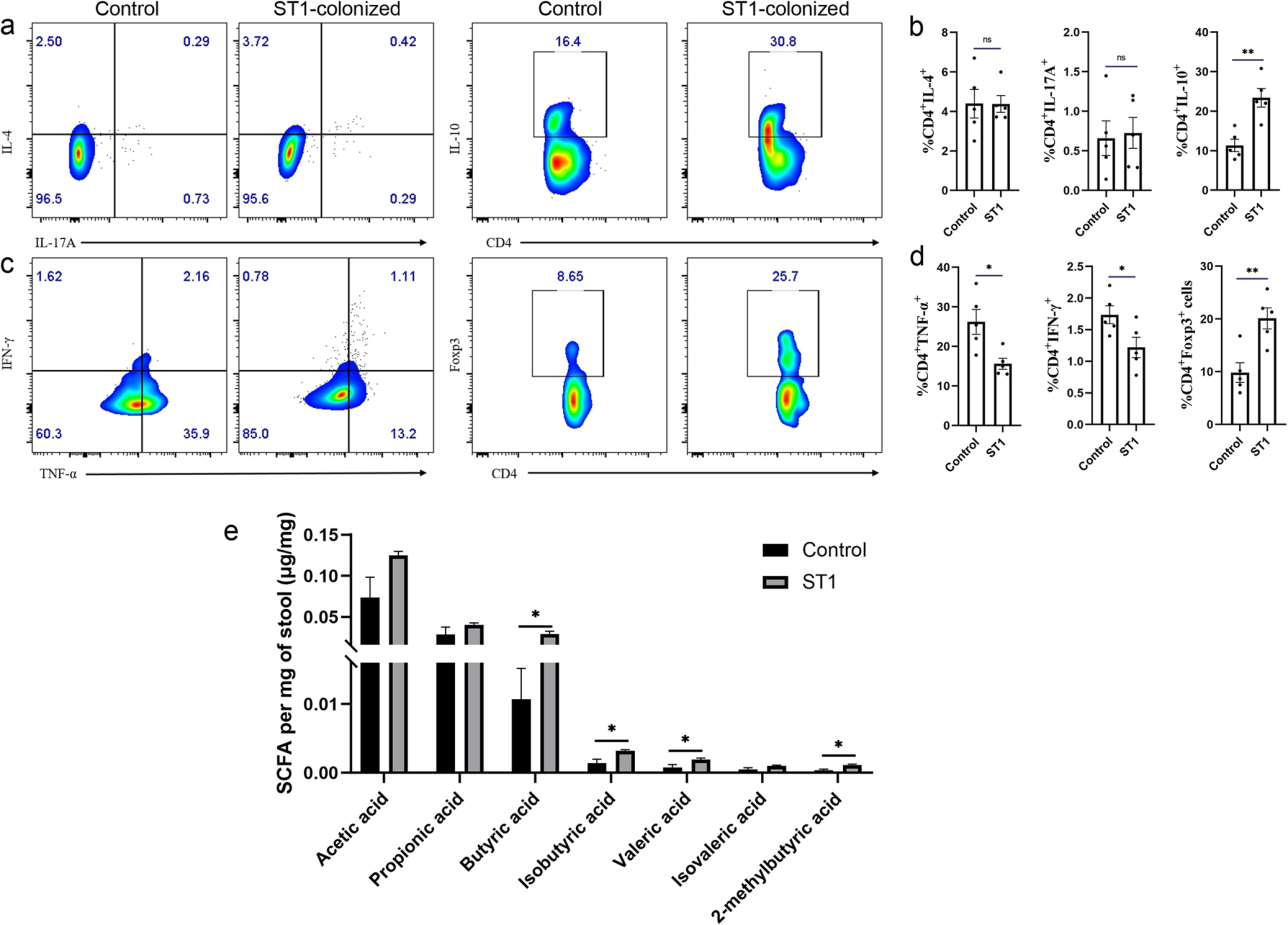
*Blastocystis* ST1-altered gut microbiota exerts beneficial effects in DSS-induced colitis mice. **a** Colored contour plots show staining for IL-4, IL-10, and IL-17A within CD4^+^ cells. **b** Bar charts show the percentage of IL-4, IL-10, and IL-17A expressing CD4^+^ T cells. **c** Colored contour plots show staining for IFN-γ, TNF-α, and Foxp3 within CD4^+^ cells. **d** Bar charts show the percentage of IFN-γ, TNF-α, and Foxp3-expressing CD4^+^ T cells. **e** SCFA concentration in mice with ST1-altered gut microbiota and control donor. Data are shown as the mean ± SEM. Significance was determined by two-sided unpaired Student’s *t* test (ns non-significant; *P < 0.05; **P < 0.01).

Since our previous study showed *Blastocystis* ST4-altered gut microbiota increased SCFA production and the number of CD4^+^ cells expressing IL-10 to suppress the DSS-induced colitis^16^, we sought to evaluate the effect of the ST1-altered gut microbiota on colonic T helper cells and SCFA production. Similarly, ST1-altered gut microbiota recipient mice showed a higher proportion of anti-inflammatory cytokine IL-10 and Foxp3^+^-Treg cells (Fig. 7a–d). In contrast, the pro-inflammatory cytokine IFN-γ and TNF-α-producing Th1 cells were increased significantly in control recipient mice (Fig. 7c, d). Interestingly, the levels of butyric, isobutyric, valeric acid and 2-methylbutyric acid in recipient mice of ST1-altered gut microbiota exhibited significantly higher concentrations compared to control mice (Fig. 7e). These findings suggest that the gut microbiota altered by *Blastocystis* ST1 have a beneficial effect on host health by regulating adaptive immune response and SCFA production.

## Discussion

The associations between *Blastocystis* and gut microbiota have been investigated in several studies, but the pathogenicity of *Blastocystis* remains controversial^4,32^. Several studies report that *Blastocystis* colonization was associated with healthy gut microbiota and the organism was thus considered a commensal^6,33,34^. On the other hand, several studies reported that *Blastocystis* could cause dysbiosis and is associated with decreased cognitive function^8,18^. In the present study, we utilized a specific subtype (ST1), which is one of the most common subtypes found in humans. We observed that ST1 colonization was positively associated with the beneficial bacteria *Alloprevotella* and *Akkermansia*, and could prevent experimentally-induced colitis through the accumulation of Treg cells.

Alterations to the gut microbiota are implicated in the pathogenesis of IBD^25^. *Alloprevotella* is generally considered to be an SCFA producer, and the decreased abundance of *Alloprevotella* was correlated with reduced acetic acid and propionic acid concentrations in a mouse model^21^. Moreover, a positive correlation was observed between *Alloprevotella* and the anti-inflammatory cytokine IL-10 in mice with DSS-induced colitis, indicating a potential link to the protective effect against inflammation^22^. The increased level of *Alloprevotella* and other taxa is associated with reduced intestinal inflammation^35^. Multiple studies have shown that *Akkermansia* is associated with a healthy intestinal condition^36–38^. It has been determined that administration of *Akkermansia muciniphila* can ameliorate experimentally caused colitis^23^, and a lower proportion of *Akkermansia* has been identified in both fecal samples and mucosal biopsies of ulcerative colitis patients^39,40^. *A. muciniphila* can adhere to human colonic cell lines (Caco-2 and HT-29) and enhance barrier function in vitro, suggesting its ability to strengthen the compromised intestinal barrier^41^. Besides, *A. muciniphila* can also induce T-cell immune responses during homeostasis^42^, and increased abundance was observed in time-restricted feeding rats^43^. Although a previous study showed ST4 colonization increases *Akkermansia* level^7^, this is the first study to show a causal relationship between *Blastocystis* colonization and *Akkermansia* levels.

Previous studies had indicated that *Blastocystis* ST1 is involved in host innate immune responses. For example, co-incubation of ST1 with the cell lines HT-29 and T-84 increased the release of IL-8 and GM-CSF^44^. ST1 can also induce intestinal epithelial cells to secrete antimicrobial peptides (LL-37)^45^. The present study is the first study to show that *Blastocystis* ST1 can activate Th2 and Treg cell immune responses to prevent mice from DSS-induced colitis. Th2 cells are involved in type 2 immune responses, which are important for the eradication of extracellular parasites and bacterial infection^46^. Th2 cells were shown to suppress INF-γ-producing Th1 cells to inhibit pro-inflammatory immune responses^47^. Gut microbiota has been shown to induce intestinal Foxp3^+^ Treg cells and to enhance the expression of IL-10 in Treg cells^48^. IL-10 can suppress the secretion of pro-inflammatory cytokines, such as TNFα, to improve colonic inflammation in the DSS-induced colitis model^49^. IL-10 knockout mice are prone to develop spontaneous intestinal colitis, suggesting it is an essential immunoregulator in the intestinal tract^50^. Secretion of the anti-inflammatory cytokine IL-10 can suppress Th1 and Th17 effector functions, both of which are key mediators of IBD^51^.

*Blastocystis* ST1 strains exhibit extensive genetic variations, suggesting that this subtype may exert strain-dependent differential effects on host health^52^. For example, *Blastocystis* ST1 was regarded as a pathogenic subtype since it was significantly associated with IBS Diarrhea (IBS-D) in Indonesian adolescents^53^. *Blastocystis* ST1 was also the most predominant subtype in colorectal carcinoma (CRC) patients and has a strong significant association risk with CRC^54^. Besides, ST1 isolated from a symptomatic patient led to lethargy and intestinal inflammation in rats^55^. On the other hand, ST1 isolated from asymptomatic human donors did not result in any clinical symptoms and can colonize asymptomatically for more than one year^56^, and numerous studies revealed that ST1 mainly colonized healthy individuals^19^. Our data showed that ST1 isolated from a healthy individual during health screening in NUH did not cause any pathology in mice colon tissues, and instead, ST1 colonization played a protective role in the development of DSS-induced colitis. However, the effects of ST1 on the host should be expanded to different ST1 isolates to better explain its exact effects on host health.

While our study demonstrated that colonization with *Blastocystis* ST1 led to significant changes in the gut microbiota and altered host susceptibility to DSS-induced colitis, the precise mechanism behind this effect is still unclear. The effect may be mediated through direct interaction between ST1 and the gut bacteria/archaea, leading to changes in the microbial community structure and function. Alternatively, ST1 could be producing metabolites that affect the gut microbiota and host immune system. Future studies using targeted metagenomic and metabolomic analyses could help shed light on the specific mechanisms underlying the effect of ST1 on the gut microbiota and host health.

The germ-free mouse model is an important tool for understanding the complex interactions between microorganisms and host health, and has been used to investigate the role of microorganisms in various diseases, including IBD^57^. Using this mouse model would eliminate the confounding factors associated with the presence of other microorganisms in the gut, providing a clear picture of the impact of *Blastocystis* ST1 on the gut microbiota and the host immune response. Besides, adding a non-*Blastocystis* colonized control microbiota along with *Blastocystis* to see if the effect on the host could be “rescued,” could allow for a more targeted investigation of the role of *Blastocystis* on the host.

**Fig. 8:**
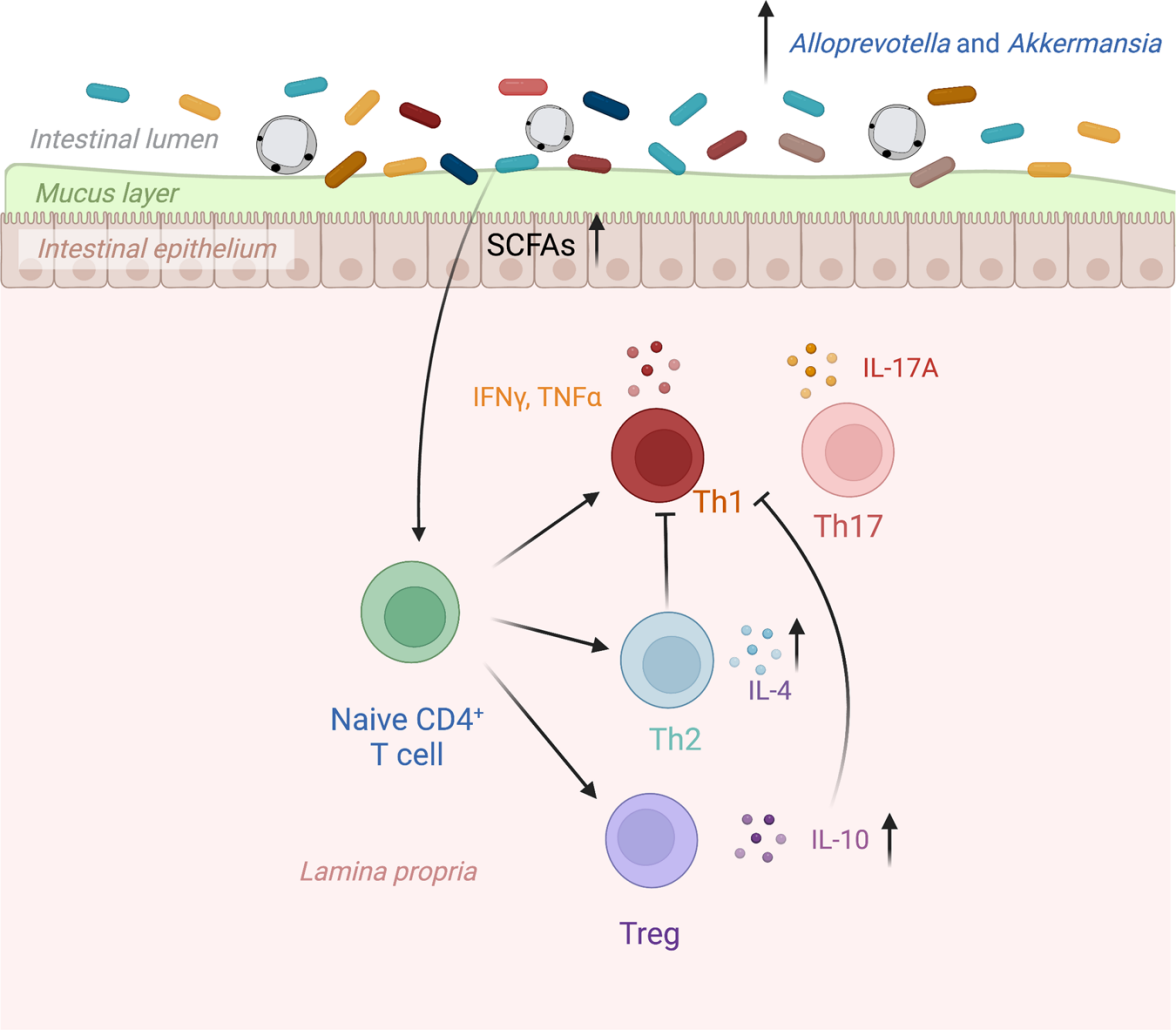
Proposed model for *Blastocystis* ST1 colonization on intestinal health. *Blastocystis* ST1 alters intestinal microbiota composition resulting in increases of beneficial bacteria *Alloprevotella* and *Akkermansia*. These species are associated with modulation of intestinal inflammation through Th2 and Treg immune responses, which suppress Th1-associated inflammation. ST1 mediates increases in SCFA in colonized mice, through increases in SCFA producers, which also play a role in suppressing inflammation through Treg responses.

In conclusion, we report that *Blastocystis* ST1 colonization in a mouse model was associated with a higher proportion of the beneficial bacteria *Alloprevotella* and *Akkermansia*, and induced protective immune responses. Furthermore, ST1-colonized mice and recipient mice of ST1-altered gut microbiota were refractory to DSS-induced colitis via increasing the anti-inflammatory cytokine IL-10 and SCFA production, indicating that *Blastocystis*-mediates healthy gut physiology through positive alteration in the microbiota bacterial flora. A summary of these findings is illustrated in Fig. 8.

## Methods

### Culture of *Blastocystis*

ST1-NUH9 was originally isolated in 2007 from a patient undergoing routine screening at the National University Hospital^58^. Permission from the National Healthcare Group Institutional Review Board was given before project commencement. Axenization was performed to obtain purified *Blastocystis* isolates^59^. Briefly, ST1-NUH9 isolate was cultured in Jones’ medium under aerobic conditions for 7 days, and then inoculated into pre-reduced Iscove’s Modified Dulbecco’s Media (IMDM) supplemented with 10% horse serum (Gibco) and antibiotics (2000 μg/ml claforan, 500 μg/ml ampicillin, 100 μg/ml streptomycin, and 100 UI/ml penicillin). Cultures were subcultured every 7 days until *Blastocystis* cells equaled or outnumbered bacterial ones. *Blastocystis* was then grown in 3.6% Bacto Agar, and *Blastocystis* colonies differentiated from bacterial ones by microscopy. *Blastocystis* colonies were isolated and transferred into pre-reduced IMDM supplemented with horse serum and antibiotics to obtain purified *Blastocystis* cells, using a Pasteur pipette. Axenized *Blastocystis* ST1 was maintained in 10 ml of pre-reduced IMDM supplemented with 10% heat-inactivated horse serum. Cultures were incubated under anaerobic conditions in an AnaeroJar (Oxoid) with a gas pack (Oxoid) at 37 °C. The number of *Blastocystis* cells was manually calculated using a hemocytometer (Kova International). Before each experiment in which it was included as an inoculum, the *Blastocystis* ST1 isolate was assessed for purity through microscopy. For quality control, supernatants from *Blastocystis* cultures were plated on Trypticase soy agar with defibrinated sheep blood (BD) to observe for bacteria colonies.

### Mice and treatments

C57BL/6 mice, aged 8–12 weeks, were maintained in isolated ventilated IsoCages in the animal facility of the National University of Singapore (NUS). Littermates of the same sex and age were randomly assigned to different experimental groups. Mice were orally gavaged with 5 × 10^7^ live *Blastocystis* ST1 cells^16^. In total, 2% DSS (MP Biomedicals) in drinking water was administrated to mice to induce acute colitis. Mice were administrated with a broad-spectrum antibiotic cocktail (0.5 g/L of vancomycin and 1 g/L of ampicillin, neomycin, and metronidazole), before the microbiota transplant experiments^60^. The disease activity index (DAI), assessed by weight changes, stool consistency, and the presence of fecal blood, was used to determine the severity of colitis^61^. All animal experiments were performed under the Singapore National Advisory Committee for Laboratory Animal Research guidelines and approved by the Institutional Animal Care and Use Committee of NUS (R19-1259).

### Fecal microbiota transplantation

Fresh fecal samples were collected from ST1-colonized and non-colonized mice and frozen at –80 °C for 1 week. The live *Blastocystis* ST1 cells were ruptured by freeze-thaw to exclude their effect on experimentally caused colitis. The presence or absence of live *Blastocystis* cells was determined by culturing the feces in Jones’ medium (Supplementary Fig. 2). Fecal microbiota transplantation (FMT) was performed by diluting frozen feces with pre-reduced PBS (50 mg/ml) and administering the mixture to recipient mice via oral gavage (10 mg/mice) three times a week^62^.

### Scanning electron microscopy

Scanning electron microscopy (SEM) was performed to determine the colonization status of *Blastocystis* ST1^63^. The cecum/colon tissues were cut longitudinally without disturbing the intestinal contents. The opened tissues were pinned down to a silicone mat and fixed in 2.5% glutaraldehyde at 4 °C overnight. The tissues were washed with PBS and processed by post-fixing in 1% osmium tetroxide for 1 h, followed by dehydration with increasing concentrations of ethanol and critical-point drying. The dried samples were coated with 25 nm gold and imaged on a field emission JSM-6701F SEM at a voltage of 10 kV.

### DNA extraction and real-time quantitative PCR

Genome DNA from fecal samples was extracted using QIAamp Fast DNA Stool Mini Kit (Qiagen) according to the manufacturer’s protocol. Real-time quantitative PCR (qPCR) was used to estimate the number of *Blastocystis* cells in fecal samples^64^. The extracted DNA was combined with a mixture of 5 µl of SsoAdvanced Universal SYBR Green Supermix, along with 0.5 μM forward primer BL18SPPF1 (5′- AGTAGTCATACGCTCGTCTCAAA-3′) and 0.5 μM reverse primer BL18SR2PP (5′-TCTTCGTTACCCGTTACTGC-3′) specific for the SSU rRNA gene of *Blastocystis*. The quantification of *Blastocystis* was carried out on an ABI 7500 real-time PCR system instrument (Life Technologies) with the following thermal cycling conditions: initial denaturation at 95 °C for 5 min, followed by 45 cycles of 95 °C for 15 s, 68 °C for 10 s, and 72 °C for 15 s. ST1-NUH9 cultures and IMDM medium were used as positive and negative control, respectively.

### Colon histology

Colonic tissues were collected at the end of experiments and fixed in 4% neutral buffered formalin, and then processed and embedded in paraffin. Sections of 4.5 μm were prepared and stained with hematoxylin and eosin (H&E). Histology scoring was performed in a blinded fashion, whereby changes in intestinal crypt architecture, degree of inflammation, and epithelium damage were scored^65^.

### 16 S rRNA gene sequencing and bioinformatics analysis

The 16 S rRNA gene sequencing library was constructed using a MetaVX Library Preparation Kit (South Plainfield, NJ). Briefly, 20–30 ng of DNA was used to generate amplicons that cover V3 and V4 hypervariable regions of the 16 S rRNA gene of bacteria. The forward primer contains the sequence ‘CCTACGGRRBGCASCAGKVRVGAAT’, and the reverse primers contain the sequence ‘GGACTACNVGGGTWTCTAATCC’. The 25 µl PCR mixture was prepared with 2.5 µl TransStart buffer, 2 µl dNTPs, 1 µl of each primer, 0.5 µl TransStart Taq DNA polymerase, and 20 ng template DNA. The PCR was performed with the following program: 3 min of denaturation at 94 °C, 24 cycles of 5 s at 95 °C, 90 s of annealing at 57 °C, 10 s of elongation at 72 °C, and a final extension at 72 °C for 5 min. Indexed adapters were added to the ends of the amplicons by limited cycle PCR. Finally, the library was purified using magnetic beads. DNA concentration was determined with a microplate reader (Tecan, Infinite 200 Pro) and the fragment size was determined by 1.5% agarose gel electrophoresis, which was expected at ~600 bp. Next-generation sequencing was conducted on an Illumina Novaseq Platform (Illumina, San Diego, USA) at the laboratory. Automated cluster generation and 250 paired-end sequencing with dual reads were performed according to the manufacturer’s instructions.

Paired-end sequences of positive and negative reads were filtered, denoised, and chimeras removed to obtain amplicon sequence variants (ASVs) through DADA2 using the Quantitative Insights into Microbial Ecology 2 (Qiime2) plugin^66^. The Silva 138 database was used for the taxonomic analysis of the representative ASV sequences^67^. Shannon and Chao1 were used to estimate the bacterial diversity and richness respectively. Beta diversity was assessed by permutational multivariate analysis of variance (PERMANOVA). Principal coordinates analysis (PCoA) plots were constructed based on Bray–Curtis dissimilarity to illustrate the differences in community structure between different groups. Heatmaps were used to show the different taxa between groups. Linear discriminant analysis (LDA) effect size (LEfSe) analysis was performed to detect bacterial taxa with significantly different abundance among different groups with *P* value <0.05 and LDA score >4 (https://huttenhower.sph.harvard.edu/galaxy/).

### Isolation of colon lamina propria cells

To analyze colonic lymphocytes, the colon tissues were longitudinally opened and washed with ice-cold PBS to remove luminal contents. The tissues were cut into 1 cm pieces and incubated in Roswell Park Memorial Institute (RPMI) 1640 medium (Sigma-Aldrich) containing 1 mM EDTA (Sigma-Aldrich) and 1 mM DTT (Sigma-Aldrich) at room temperature for 20 min under slow rotation and spun down to remove the supernatant. The remaining tissue pieces were incubated in RPMI containing 25% HEPES, 10% fetal calf serum (FCS), 1 mM EDTA, and 1 mM DTT at 37 °C for 1 h under slow rotation and then washed by PBS to remove epithelial cells and intraepithelial lymphocytes. Tissue pieces were digested with Liberase (Sigma-Aldrich) at 37 °C for 30 min under slow rotation. The digested tissue pieces and supernatants were filtered with 70-μm cell strainers and glass wool separately. After centrifugation, pellets containing the lamina propria (LP) lymphocytes were harvested.

### Flow cytometry analysis

Lymphocytes were stimulated for 6 h with a cell stimulation cocktail of PMA (50 ng/ml), ionomycin (750 ng/ml), and GolgiStop (monensin, BD Biosciences). Live/dead stain was used to evaluate the viability of the cells using live/dead fixable viability stain kits. For surface staining, stimulated cells were stained with TCR beta monoclonal antibody (BUV510, eBioscience), and anti-CD4 (BUV395; Biolegend). Fixation and permeabilization buffers (Biolegend) were used for intracellular cytokine staining. Fixed and permeabilized cells were stained with fluorochrome-conjugated anti-mouse antibodies against IL-4 (BUV421; Biolegend), IL-10 (PE; Biolegend), IL-17A (APC; eBioscience), interferon-gamma (IFN-γ) (BUV711; Biolegend), tumor necrosis factor (TNF-α) (APC; eBioscience), and Foxp3 (PE, Biolegend) at 4 °C for 10 h. Flow cytometry analysis was performed on a Fortessa X-20 (BD biosciences) flow cytometer and the data were analyzed using FlowJo V10 software. Gating strategy of immune populations in the colonic LP was shown in Supplementary Fig. 3.

### LC/MS/MS assay

Liquid chromatography/tandem mass spectrometry (LC/MS/MS) was performed for analysis of short-chain fatty acid (SCFA) in derivatized stool extracts^68^. Briefly, 500 μl of ice-cold extraction solvent containing 10 μM of d_5_-benzoic acid as internal standard (IS) was added to 250 mg of stool samples. The mixture was then spun down, and the supernatant was discarded. Subsequently, an aliquot of 100 μl was derivatized using aniline and EDC. The derivatization reaction was quenched using succinic acid and 2-mercaptoethanol. All samples were saved at 4 °C until analysis on the same day. Analysis was carried out using an Agilent 1290 Infinity LC system (Agilent Technologies, Santa Clara, CA, USA).

### Statistical analysis

Statistical analysis was performed in Prism 8 (Graphpad Software Inc.). Two independent replicates were performed for each experiment. A two-sided unpaired Student’s *t* test was used for comparisons of two groups. Error bars on graphs display the mean and SEM. *P* values of <0.05 were considered significant; the following symbols were used to indicate significance levels: ns, non-significant; *P < 0.05; **P < 0.01; ***P < 0.001; and ****P < 0.0001.

## Supplementary information

Supplementary Information

## Data Availability

The datasets generated and analyzed in the current study are available in the Sequence Read Archive (SRA) database at NCBI under BioProject ID PRJNA891885. All other data are available from the corresponding author upon request.
