# Supplementary material for: Colonization with ubiquitous protist Blastocystis ST1 ameliorates DSS-induced colitis and promotes beneficial microbiota and immune outcomes
Source: NPJ Biofilms Microbiomes. 2023 Apr 25;9:22. doi: 10.1038/s41522-023-00389-1 (PMC10130167; doi:10.1038/s41522-023-00389-1)
Supplement: Supplementary file 1 — Supplementary Information [file 41522_2023_389_MOESM1_ESM.pdf]

Supplementary materials for

Colonization with ubiquitous protist *Blastocystis* ST1 ameliorates DSS-induced colitis and promotes beneficial microbiota and immune outcomes

Lei Deng[1], Lukasz Wojciech[2], Chin Wen Png[1], Yan Qin Dorinda Kioh[3], Geok Choo Ng[1], Eric Chun Yong Chan[3], Yongliang Zhang[1], Nicholas R. J. Gascoigne[2], and Kevin Shyong Wei Tan[1,*]

[1] Laboratory of Molecular and Cellular Parasitology, Healthy Longevity Translational Research Programme and Department of Microbiology and Immunology, Yong Loo Lin School of Medicine, National University of Singapore, 5 Science Drive 2, Singapore 117545, Singapore

[2] Department of Microbiology and Immunology, Immunology Translational Research Programme, Yong Loo Lin School of Medicine, National University of Singapore, 117545 Singapore

[3] Department of Pharmacy, Faculty of Science, National University of Singapore, 5 Science Drive 2, Singapore 117545, Singapore

* Correspondence: mictank@nus.edu.sg

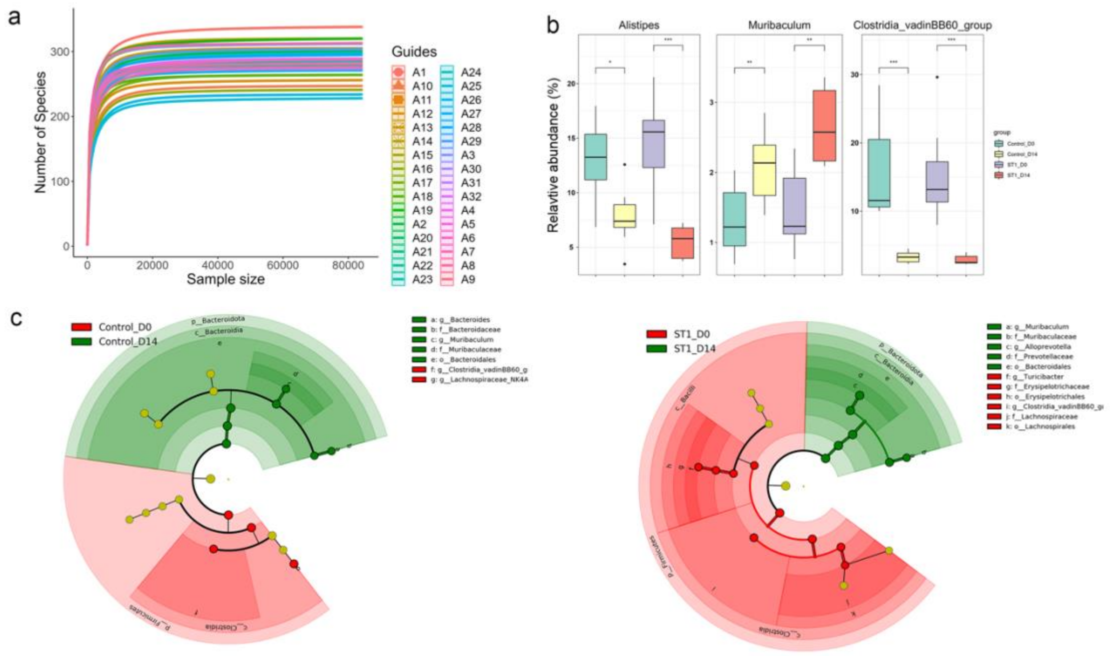

Supplementary Figure 1. **The colonization of *Blastocystis* ST1 leads to alterations in the composition of gut microbiota. a** Rarefaction curves. **b** Significant differences of genera in *Blastocystis*-free and ST1-colonized mice. Data are presented as boxplots, with the center line representing the median, the boundary of the whiskers representing the minimum and maximum values of the dataset, and the boundary of the box representing the 25th and 75th percentile of the dataset. **c** Cladogram generated by LEfSe indicating differentially abundant bacterial taxa in ST1-colonized and control mice when compared to baseline

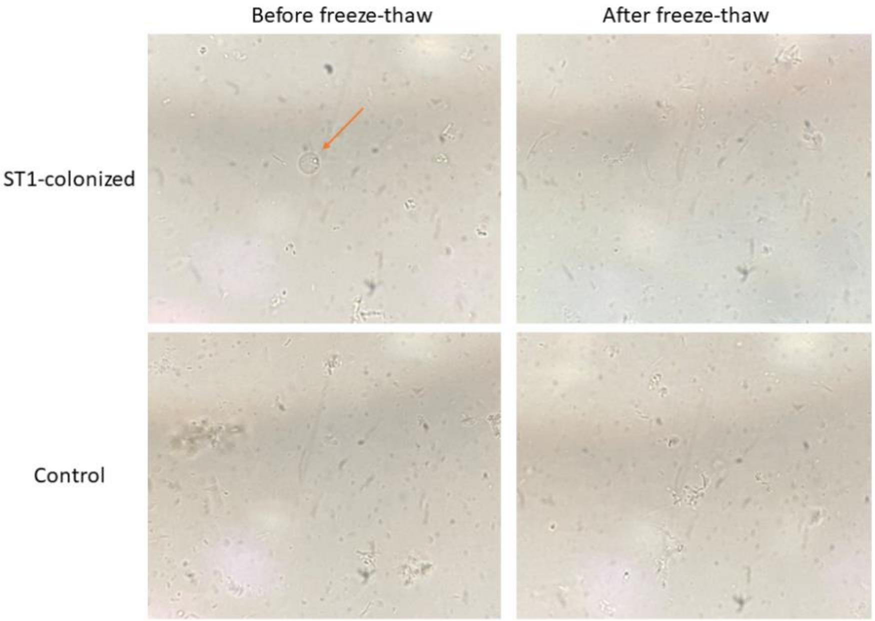

Supplementary Figure 2. The feces from ST1-colonized and control mice cultured in Jones' medium.

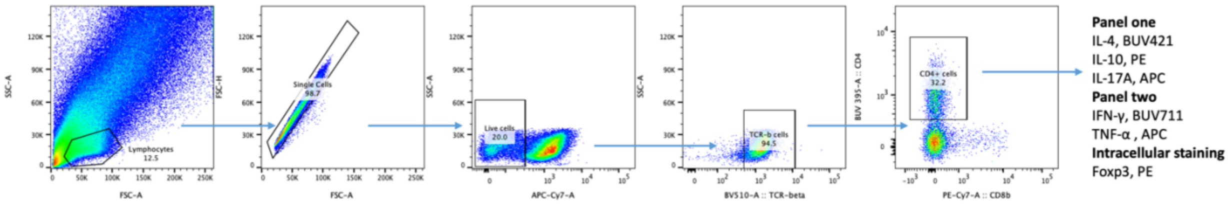

Supplementary Figure 3. **Gating strategy of immune populations in the colonic lamina propria.**

Supplementary Table 1. PERMANOVA of beta-diversity analysis as measured by Bray-Curtis dissimilarity among different groups.

**pairs**	**Df**	**SumsOfSqs**	**F.Model**	**R2**	**p.value**	**p.adjusted**	**sig**
Control_D0 vs ST1_D0	1	0.058373	0.650173	0.04438	0.84	0.84	
Control_D0 vs Control_D14	1	0.537656	6.899169	0.330117	0.001	0.0012	*
Control_D0 vs ST1_D14	1	0.617413	8.688156	0.382938	0.001	0.0012	*
Control_D14 vs ST1_D0	1	0.486671	6.716525	0.324211	0.001	0.0012	*
Control_D14 vs ST1_D14	1	0.304711	5.669878	0.288252	0.001	0.0012	*
ST1_D0 vs ST1_D14	1	0.549864	8.383098	0.374528	0.001	0.0012	*
